# Supplementary material for: Crystallographic and cryogenic electron microscopic structures and enzymatic characterization of sulfur oxygenase reductase from Sulfurisphaera tokodaii
Source: J Struct Biol X. 2020 Jul 3;4:100030. doi: 10.1016/j.yjsbx.2020.100030 (PMC7398979; doi:10.1016/j.yjsbx.2020.100030)
Supplement: Supplementary data 1 [file mmc1.docx]

**Supplementary Materials**

**Crystallographic and cryogenic electron microscopic structures, and enzymatic characterization of sulfur oxygenase reductase from *Sulfurisphaera tokodaii***

**Contains:**

- **Supplementary Table S1-S2**
- **Supplementary Figures S1-S9**
- **Supplementary Methods**
  - **Cryo-EM data processing**
  - **Model refinement and analysis of the cyro-EM map without imposing symmetry**
- **References**

**Table S1.** Predicted enzymes in the genome of *S. tokodaii* strain 7.

| Locus tag | Length (a.a.) | Predicted enzyme name  (EC number) | Predicted function |
| --- | --- | --- | --- |
| STK_RS02900 | 374 | Cystathionine gamma-synthase  (EC 2.5.1.48) | *O*-succinyl-L-homoserine + L-Cys →L-cystathionine + succinate |
| STK_RS03465 | 133 | Rhodanese-like  (EC 2.8.1.1) | S_2_O_3_^2–^ + CN^–^ ↔ SO_3_^2–^ +SCN^–^  S^0^, S_2_O_3_^2–^ → S^0^_(n)_ |
| STK_RS05385 | 390 | Sulfide dehydrogenase flavoprotein subunit (EC 1.8.1.19) | H_2_S + S^0^_(n)_ + NADP^+^→S^0^_(n+1)_ + NADPH + H^+^ |
| STK_RS04570 | 200 | Sulfite oxidase  (EC 1.8.3.1) | SO_3_^2–^ + O_2_ + H_2_O → SO_4_^2–^ + H_2_O_2_ |
| STK_RS06245  (StSOR) | 311 | Sulfur oxygenase reductase  (EC 1.13.11.55) | 5S^0^ + O_2_ + 4H_2_O → SO_3_^2–^ + S_2_O_3_^2–^ + 2H_2_S + 4H^+^ |
| STK_RS07750 | 300 | Cysteine synthase  (EC 2.5.1.47) | *O*-acetyl-L-serine + H_2_S →L-Cys + acetate |
| STK_RS09980 | 267 | Rhodanese (sulfurtransferase)  (EC 2.8.1.1) | S_2_O_3_^2–^ + CN^–^ ↔ SO_3_^2–^ +SCN^–^ |
| STK_RS10330  STK_RS10335 | 180  168 | Thiosulfate: quionone oxidoreductase  (EC 1.8.5.2) | 2S_2_O_3_^2–^ + quinone + 2H^+^ ↔ S_4_O_6_^2–^ + quinol |
| STK_RS14050 | 293 | Rhodanese (sulfurtransferase)  (EC 2.8.1.1) | S_2_O_3_^2–^ + CN^–^ ↔ SO_3_^2–^ +SCN^–^ |
| STK_RS14060 | 628 | Sulfite reductase (assimilatory?)  (EC 1.8.1.2) | H_2_S + 3Fd^ox^ + 3H_2_O ↔ SO_3_^2–^ +3Fd^red^ |
| STK_RS14065 | 239 | Phosphoadenylyl-sulfate reductase  (EC 1.8.4.8) | PAPS + Trx^red^ ↔ ABP + Trx^ox^ + SO_3_^2–^ |
| STK_RS14070 | 412 | Sulfate adenylyltransferase  (EC 2.7.7.4) | ATP + SO_4_^2–^ → PPi + APS |

Putative adenylyl-sulfate kinase gene (APS + ATP → PAPS + ADP) wad not found. The second function of rhodanese (STK_ RS03465) is based on the previous report of *A. aeolicus* rhodanese (Aq-477) (Aussignargues et al., 2012). Abbreviations: Fd, ferredoxin; PAPS, 3′-phosphoadenosine-5′-phosphosulfate; Trx, thioredoxin; ABP, adenosine 3',5'-bisphosphate; PPi, pyrophosphate; APS, adenosine 5'-phosphosulfate.

**Table S2.** High resolution single-particle cryo-EM structures in the databases with data collected by 200 kV instruments.

| Resolution (Å) | Sample | Source organism | Oligomeric state | Theoretical weight (MDa) | EMDB ID | PDB ID | Citation |
| --- | --- | --- | --- | --- | --- | --- | --- |
| 1.75 | Apoferritin | Mouse | 24 | 0.505 | 21024 | 6v21 | bioRxiv 855643 |
| 2.01 | Apoferritin | Mouse | 24 | 0.44 | 9914 | none | (Danev et al., 2019) |
| 2.13 | Aldolase | Rabbit | 4 | 0.15 | 21023 | 6v20 | bioRxiv 855643 |
| 2.24 | StSOR | *S. tokodaii* | 24 | 0.55 | 30073 | 6m3x | This work |
| 2.32 | HemQ | *Geobacillus* sp. | 5 | 0.144 | 21373 | 6vsa | bioRxiv 798280 |
| 2.56 | Capsid protein VP1 | Adeno-assisted virus | 60 | 5.08 | 20693 | 6u95 | (Kaelber et al., 2020) |

Prepared based on database searches on 21 February 2020.

**Figure S1.** Cryo-EM data processing workflow. A detailed description is given in Supplementary Methods section. Mask diameter in Å and octahedral symmetry is abbreviated MD and Oct, respectively.

**Figure S1.** (continued)

**Figure S2.** SDS-PAGE of purified recombinant samples. SDS-PAGE of purified protein samples for biochemical (A) and structural (B) characterizations are shown. (A) 1, marker; 2, crude cell extract; 3, after Q-Sepharose; 4, after Hydroxyapatite; 5, after Mono Q; 6, after Superdex 200pg. (B) 1 and 4, marker; 2, after Superdex 200pg that was used for cryo-EM; 3, after Mono Q that was used for crystallization.

**Figure S3.** Effects of inhibitors, metals, and denaturants. (A) Effect of inhibitors and metal ions. The activities in the presence of indicated concentrations (mM) of each additive were measured. Metal ions were added as chloride salts. Abbreviations: DTT, dithiothreitol; GdmCl, guanidium chloride; NEM, *N*-ethylmaleimide; *p*CMB, *p*-chloromercuribenzoate; IAA, iodoacetic acid; EDTA, ethylenediaminetetraacetic acid. Relative activities in the presence of 0.1 and 1.0 mM Zn^2+^ were 318% and 252%, respectively. (B) Stability against denaturants. Remaining activities after incubation for 30 min at 4 °C in the presence of each denaturants were measured. Symbols: filled bars, oxygenase activity; open bars, reductase activity.

**Figure S4.** Spectral analyses of purified StSOR. (A) UV-visible absorption spectra in 10 mM Tris-HCl (pH 8.0) at room temperature (25 °C). Protein concentrations are indicated. (B) CD spectrum of 0.25 mg/mL StSOR in 10 mM Na-phosphate buffer (pH 7.0) at room temperature.

**Figure S5.** Metal (Fe)-ligand (N or O) distance distribution of crystallographic (A) and cyro-EM (B) structures analyzed by CheckMyMetal server. Distance distributions in the StSOR structures (user, red bars) and Cambridge Structural Database (CSD, blue lines) are shown.

**Figure S6.** Validation of single particle analysis of StSOR. (A) The sharpened density map of the final reconstruction with the resolution of 2.24Å (0.143 FSC criterion) and the angular distributions. (B) The Fourier Shell Correlation (FSC) curves between half-sets (black) and map-to-model (green).

**Figure S7.** Cryo-EM structure of cis-peptides. Density maps of Gly111–Pro112 (A), Arg244–Pro245 (B), and Gly287–Pro288 (C) are shown with a contour level of 0.0435 (4.5σ).

**Figure S8.** Examination of the cryo-EM map refined without imposing symmetry. Zoom-in-views of the C1 map around the residues of interest with a contour level of 0.0435 (4.5σ). Representative chains selected from 24-mer chains (from “Chain A” to “Chain X”) based on variations of the potential shapes are shown. (A) Iron center. (B-D) Cys31, Cys101, and Cys104. (E) Residues forming the chimney.

**Figure S9.** Packing effects and temperature factors of the crystal structure. (A) Packing interaction of chimneys. Chain A (green) and B (cyan) of the original model coordinates and symmetry-related molecules are shown. Chains E and F in symmetry molecule 1, chains C, D, and G in molecule 2, and chain H in molecule 3 are shown in white, marine, and blue, respectively. (B and C) The 24-mer structure of StSOR colored by B-factor as a rainbow color from blue (20 Å^2^) to red (60 Å^2^). Panel (B) shows the molecular surface with no clipping, and panel (C) shows inside of the hollow sphere by clipping with 80 Å slab.

**Supplementary Methods**

**Cryo-EM data processing**

The movie frames were aligned, dose-weighted, and averaged using Motioncor2 (Zheng et al., 2017), on 5 × 5 tiled frames with a B-factor of 200 applied, to correct for beam-induced specimen motion and to account for radiation damage by applying an exposure-dependent filter. The micrographs whose total accumulated motion was larger than 100 Å were discarded. The non-weighted movie sums were used for Contrast Transfer Function (CTF) estimation (512-pixel box size, 30 Å minimum resolution, 3 Å maximum resolution, 0.10 amplitude contrast) with CTFFIND4 (Rohou and Grigorieff, 2015) and Gctf program (Zhang, 2016), while the dose-weighted sums were used for all subsequent steps of image processing. The images whose CTF max resolution was better than 5.5 Å were selected. In addition, micrographs showing obvious indication of ice crystallization (i.e. strong ice ring in the Fourier space) were manually discarded.

As shown Fig. S1, the particles were picked fully-automatically using SPHIRE-crYOLO with the generalized model (Moriya et al., 2017; Wagner et al., 2019) with 231-pixel box size and the selection threshold of 0.05. The micrographs that contain less than 36 picks were excluded. A stack of 305,182 particle images was extracted from 2,387 dose-weighted sum micrographs while rescaling to 2.76 Å/pixel with 96-pixel box size, and subjected to consecutive two runs of reference-free 2D classification (1^st^ run: 200 expected classes, 188 Å mask diameter; 2^nd^ run: 200 expected classes, 166 Å mask diameter) using RELION-3 (Zivanov et al., 2018). The 160,571 particles corresponding to the best 29 classes of 2^nd^ run that displayed secondary-structural elements and multiple views of StSOR were selected for RELION-3 *ab initio* reconstruction (asymmetry, single expected class, 188 Å mask diameter), and 176,437 particles are selected with more relaxed criteria for the subsequent RELION-3 3D classification (2 expected classes). The generated *ab initio* map was imposed octahedral symmetry, low-pass filtered to 20 Å, and used as an initial model for the 3D classification. The 3D class obviously consisted from bad images containing non-targeted objects and was removed, and the volume of the best 3D class was low-pass filtered to 15 Å, and used as an initial model for the subsequent RELION-3 3D refinement (octahedral symmetry, 240 Å mask diameter, with padding) with the 155,333 selected particles.

The second part of Fig. S1 shows the rest of the workflow. The refined volume was then rescaled to 0.69 Å/pixel with 400-pixel box size, low-pass filtered to 15 Å, and used for the subsequent 3D refinement. Accordingly, the selected particle images were also re-centered and re-extracted using the same rescale settings. The particle images which became duplicated as the result of alignments and not fully inside of the micrograph boundary anymore due to changing the box size were excluded. 152,484 selected particles were 3D auto-refined (octahedral symmetry, 240 Å mask diameter, no padding) twice, the 1^st^ run without 3D mask and the 2^nd^ with a soft-edged 3D mask created from the initial 3D reference of 1^st^ run (5-pixel extension, 10-pixel soft cosine edge). The 2^nd^ run resulted in the resolution of 2.88 Å. 152,457 particles were again re-centered and re-extracted without changing the rescale settings, followed by removal of duplicated and out-of-bounds particles. To refine per-particle defocus, beam tilt, and beam-induced motion corrections, the cycle of CTF refinement and Bayesian polishing (Zivanov et al., 2019) in RELION-3 was repeated four times. To measure degree of the improvement, 3D refinement (octahedral symmetry, 240 Å mask diameter, no padding) with the volume of previous run as initial 3D reference, a soft-edged 3D mask created from the initial reference (5-pixel extension, 10-pixel soft cosine edge) and solvent-flattened FSCs options was used after each CTF refinement and Bayesian polishing step. The 3D refinement after 4^th^ Bayesian polishing run yielded the resolution of 2.44 Å.

At this point, the pixel size was checked by comparing the 2.44 Å cryo-EM structure with the atomic-coordinate model (PDB code: 6M35) using “Fit in Map” tool in UCSF Chimera (Pettersen et al., 2004), and calibrated to 0.676 Å/pixel which minimized the number of “atoms outside contour”. Using the calibrated pixel size, 146,465 selected particles were again re-centered and re-extracted with 480-pixel box size from 2,387 dose-weighted sum micrographs, followed by removal of duplicated and out-of-bounds particles. Accordingly, the density map was also rescaled with the same settings, low-pass filtered to 15 Å, and used as an initial model for the subsequent 3D refinement (octahedral symmetry, 236 Å mask diameter, no padding) with a soft-edged 3D mask (5-pixel extension, 10-pixel soft cosine edge). Then, the two cycles of CTF refinement and Bayesian polishing steps were executed and generated the 2.25 Å structure.

To improve homogeneity of the particle stack, no-alignment 3D classification was conducted by setting expected classes to 2 and regularization parameter T to 16 (octahedral symmetry, 236 Å mask diameter, no padding) with a soft-edged 3D mask (5-pixel extension, 10-pixel soft cosine edge), and selected 85,621 particles by choosing the 3D class with the best resolution. The last 3D refinement (octahedral symmetry, 236 Å mask diameter, no padding) with a soft-edged 3D mask (5-pixel extension, 10-pixel soft cosine edge) and solvent-flattened FSCs option generated the final result of 2.24 Å resolution, while the model-to-map FSC resolution was 2.27 Å (Fig. S6B). The re-runs of the last Bayesian polishing step excluding various numbers of the last movie frames to adjust the total electron exposure did not improve the reconstruction. The local resolution of the final reconstruction was estimated using the RELION-3’s own implementation. To check possible differences among the 24 chains of the StSOR structure, the last 3D refinement was repeated without imposing symmetry (C1) and obtained the 2.73 Å resolution. Model refinement and a brief analysis on the C1 cryo-EM map is described below.

For calculation of the global resolution estimation after each 3D refinement, the gold-standard FSC resolution with 0.143 criterion (Rosenthal and Henderson, 2003) was used, including the phase randomization to account for the possible artifactual resolution enhancement caused by solvent mask (Chen et al., 2013). The model-to-map FSC resolution with 0.5 criterion was calculated using phenix.mtriage (Afonine et al., 2018a). For the visualization of the output 2D/3D images, UCSF Chimera and e2display.py of EMAN2 (Tang et al., 2007) were used. To calculate the smallest box size that ensures no CTF aliasing in the reciprocal space up to an expected resolution for the maximum defocus value of the dataset, the ctflimit function (Penczek et al., 2014) implemented in SPARX/SPHIRE (Hohn et al., 2007; Moriya et al., 2017) was used.

**Model refinement and analysis of the cyro-EM map without imposing symmetry**

Using the cryo-EM map reconstructed without imposing symmetry (C1 map) at 2.73 Å resolution, the protein model (without water molecules) was refined automatically with “Real-Space Refine” (Afonine et al., 2018b) in PHENIX. Water molecules were placed using Coot (Emsley et al., 2010), and further refinement was performed using Coot and “Real-Space Refine” in PHENIX. Validation of the refined model was carried out using “Comprehensive validation” in PHENIX. MolProbity score, clashscore, and rotamer outliers were 2.08, 6.14, and 4.93%, respectively. Composition of residues in favored, allowed, and outlier regions of Ramachandran plot were 96.59%, 3.26%, and 0.15%, respectively. Main chain structures of the 24 chains were virtually identical, as the average RMSD and their standard deviation for the Cα atoms between all chain pairs were 0.137 ± 0.010 Å (maximum = 0.166 Å in 276 pairs).

All chains in the C1 cryo-EM map were visually inspected at the iron center, the three cysteines in the active site pocket, and the chimney. Selected representative chains for each site are shown in Fig. S8. The potential around the iron atom in the C1 map showed slight difference in each chain, and the Wat1 molecule was missing or barely visible in all chains, likely due to the deterioration of resolution (Fig. S8A). The potential around Cys31 showed large variety, exhibiting a shorter branch (chain C), a similar shape to the octahedral symmetry-refined map (chain E), or 3-way branch (chain X, Fig. S8B). Cys101 also showed some variety with ambiguous blobs (chains E and P), while chain A showed similar potential shape to the octahedral symmetry-refined map (Fig. S8C). The side chain of Cys104 were not visible in all chains as in the octahedral symmetry-refined map (Fig. S8D). Likewise, the potentials of the side chains of the chimney exhibited similar features to the octahedral symmetry-refined map (Fig. S8E). In summary, although we could not observe significant inter-chain difference related to the flexibility of the chimney and the other regions of interest, the Wat1 molecule coordinated to the iron and the side chains of Cys31 and Cys101 exhibited subtly different features depending on chains. However, any plausible interpretations of these differences could be made from the data obtained in the current and previous studies.

**References**

Afonine, P. V., Klaholz, B.P., Moriarty, N.W., Poon, B.K., Sobolev, O. V., Terwilliger, T.C., Adams, P.D., Urzhumtsev, A., 2018a. New tools for the analysis and validation of cryo-EM maps and atomic models. Acta Crystallogr. Sect. D Struct. Biol. 74, 814–840. https://doi.org/10.1107/S2059798318009324

Afonine, P. V., Poon, B.K., Read, R.J., Sobolev, O. V., Terwilliger, T.C., Urzhumtsev, A., Adams, P.D., 2018b. Real-space refinement in PHENIX for cryo-EM and crystallography. Acta Crystallogr. Sect. D Struct. Biol. 74, 531–544. https://doi.org/10.1107/S2059798318006551

Aussignargues, C., Giuliani, M.C., Infossi, P., Lojou, E., Guiral, M., Giudici-Orticoni, M.T., Ilbert, M., 2012. Rhodanese functions as sulfur supplier for key enzymes in sulfur energy metabolism. J. Biol. Chem. 287, 19936–19948. https://doi.org/10.1074/jbc.M111.324863

Chen, S., McMullan, G., Faruqi, A.R., Murshudov, G.N., Short, J.M., Scheres, S.H.W.W., Henderson, R., 2013. High-resolution noise substitution to measure overfitting and validate resolution in 3D structure determination by single particle electron cryomicroscopy. Ultramicroscopy 135, 24–35. https://doi.org/10.1016/j.ultramic.2013.06.004

Danev, R., Yanagisawa, H., Kikkawa, M., 2019. Cryo-Electron Microscopy Methodology: Current Aspects and Future Directions. Trends Biochem. Sci. https://doi.org/10.1016/j.tibs.2019.04.008

Emsley, P., Lohkamp, B., Scott, W.G., Cowtan, K., 2010. Features and development of Coot. Acta Crystallogr. Sect. D Biol. Crystallogr. 66, 486–501. https://doi.org/10.1107/S0907444910007493

Hohn, M., Tang, G., Goodyear, G., Baldwin, P.R., Huang, Z., Penczek, P.A., Yang, C., Glaeser, R.M., Adams, P.D., Ludtke, S.J., 2007. SPARX, a new environment for Cryo-EM image processing. J. Struct. Biol. 157, 47–55.

Kaelber, J.T., Yost, S.A., Webber, K.A., Firlar, E., Liu, Y., Danos, O., Mercer, A.C., 2020. Structure of the AAVhu.37 capsid by cryoelectron microscopy. Acta Crystallogr. Sect. F Struct. Biol. Commun. 76, 58–64. https://doi.org/10.1107/S2053230X20000308

Moriya, T., Saur, M., Stabrin, M., Merino, F., Voicu, H., Huang, Z., Penczek, P.A., Raunser, S., Gatsogiannis, C., 2017. High-resolution single particle analysis from electron cryo-microscopy images using SPHIRE. J. Vis. Exp. 2017. https://doi.org/10.3791/55448

Penczek, P.A., Fang, J., Li, X., Cheng, Y., Loerke, J., Spahn, C.M.T., 2014. CTER-Rapid estimation of CTF parameters with error assessment. Ultramicroscopy 140, 9–19. https://doi.org/10.1016/j.ultramic.2014.01.009

Pettersen, E.F., Goddard, T.D., Huang, C.C., Couch, G.S., Greenblatt, D.M., Meng, E.C., Ferrin, T.E., 2004. UCSF Chimera - A visualization system for exploratory research and analysis. J. Comput. Chem. 25, 1605–1612. https://doi.org/10.1002/jcc.20084

Rohou, A., Grigorieff, N., 2015. CTFFIND4: Fast and accurate defocus estimation from electron micrographs. J. Struct. Biol. 192, 216–221. https://doi.org/10.1016/j.jsb.2015.08.008

Rosenthal, P.B., Henderson, R., 2003. Optimal determination of particle orientation, absolute hand, and contrast loss in single-particle electron cryomicroscopy. J. Mol. Biol. 333, 721–745. https://doi.org/10.1016/j.jmb.2003.07.013

Tang, G., Peng, L., Baldwin, P.R., Mann, D.S., Jiang, W., Rees, I., Ludtke, S.J., 2007. EMAN2: An extensible image processing suite for electron microscopy. J. Struct. Biol. 157, 38–46. https://doi.org/10.1016/j.jsb.2006.05.009

Wagner, T., Merino, F., Stabrin, M., Moriya, T., Antoni, C., Apelbaum, A., Hagel, P., Sitsel, O., Raisch, T., Prumbaum, D., Quentin, D., Roderer, D., Tacke, S., Siebolds, B., Schubert, E., Shaikh, T.R., Lill, P., Gatsogiannis, C., Raunser, S., 2019. SPHIRE-crYOLO is a fast and accurate fully automated particle picker for cryo-EM. Commun. Biol. 2, 1–13. https://doi.org/10.1038/s42003-019-0437-z

Zhang, K., 2016. Gctf: Real-time CTF determination and correction. J. Struct. Biol. 193, 1–12. https://doi.org/10.1016/j.jsb.2015.11.003

Zheng, S.Q., Palovcak, E., Armache, J.P., Verba, K.A., Cheng, Y., Agard, D.A., 2017. MotionCor2: Anisotropic correction of beam-induced motion for improved cryo-electron microscopy. Nat. Methods 14, 331–332. https://doi.org/10.1038/nmeth.4193

Zivanov, J., Nakane, T., Forsberg, B.O., Kimanius, D., Hagen, W.J.H., Lindahl, E., Scheres, S.H.W., 2018. New tools for automated high-resolution cryo-EM structure determination in RELION-3. Elife 7, 1–22. https://doi.org/10.7554/eLife.42166

Zivanov, J., Nakane, T., Scheres, S.H.W., 2019. A Bayesian approach to beam-induced motion correction in cryo-EM single-particle analysis. IUCrJ 6, 5–17. https://doi.org/10.1107/S205225251801463X
